# Nailfold Capillary Hemorrhages: Microvascular Risk Factors for Primary Open-Angle Glaucoma

**DOI:** 10.1155/2020/8324319

**Published:** 2020-06-08

**Authors:** Nicholas M. Pfahler, Jordan L. Barry, Indre E. Bielskus, Agni Kakouri, Michael C. Giovingo, Nicholas J. Volpe, Paul A. Knepper

**Affiliations:** ^1^Department of Ophthalmology and Visual Sciences, University of Illinois at Chicago College of Medicine, Chicago, IL, USA; ^2^Division of Ophthalmology, John H. Stroger Jr. Hospital of Cook County, Chicago, IL, USA; ^3^Department of Ophthalmology, Northwestern University Feinberg School of Medicine, Chicago, IL, USA

## Abstract

**Background:**

Primary open-angle glaucoma (POAG) is associated with systemic microvascular dysfunction including hemorrhages and other abnormalities of the nailfold capillary bed. This study aimed to verify the specificity of nailfold capillary hemorrhages and other abnormalities as risk factors for POAG.

**Methods:**

Nailfold video capillaroscopy was performed using a JH-1004 capillaroscope on the fourth and fifth digits of the nondominant hand in control (*n* = 277), POAG (*n* = 206), OHT (*n* = 57), and SG (*n* = 29) subjects. The number of hemorrhages, dilated capillaries >50 *µ*m, and avascular zones ≥200 *µ*m were counted and adjusted to counts per 100 capillaries. Descriptive analyses as well as univariate- and multivariable-adjusted logistic regression were performed comparing all groups with controls and POAG with OHT and SG. Subanalyses were conducted in POAG patients examining the association between nailfold capillary outcomes and previous glaucoma surgery, successful IOP control, or disease severity.

**Results:**

All nailfold capillary outcomes were significantly increased in POAG, no outcomes were increased in SG, and only hemorrhages were mildly increased in OHT. Hemorrhages were significantly more frequent in POAG compared with both OHT (*P* < 0.0001) and SG (*P*=0.001). There were significant trends between higher numbers of hemorrhages and POAG compared with controls, OHT, and SG, with odds ratios of 18.3 (8.5–39.4), 9.1 (1.9–13.4), and 11.8 (1.7–7.3), respectively, for the presence of two or more hemorrhages per 100 capillaries. Hemorrhages were not significantly associated with previous glaucoma surgery, successful postoperative IOP control, or disease severity in POAG.

**Conclusions:**

These findings suggest that systemic microvascular dysfunction is frequent in POAG and occurs early in the disease process. The high specificity of nailfold hemorrhages makes them viable clinical risk factors for POAG.

## 1. Introduction

Primary open-angle glaucoma (POAG) is a leading cause of irreversible vision loss worldwide. The disease is characterized by progressive excavation of the optic disc with corresponding visual field defects due to loss of retinal ganglion cells [1]. POAG has no known cause, is frequently asymptomatic, and may not be discovered until late in the disease process. Elevated intraocular pressure (IOP) is the most important risk factor for POAG and can be well controlled in most patients with topical medications or surgical intervention. Some POAG patients, however, do not exhibit increased IOP, i.e., normal tension glaucoma (NTG), and others progress despite all current treatment modalities. Non-IOP risk factors for POAG are primarily vascular in nature and include altered systemic blood pressure and reduced ocular blood flow. Systemic manifestations associated with POAG include diabetes [2], systemic hypertension [3], vasospasm [4], vascular dysfunction [5], and microvascular disease [6]. Numerous biomarkers relating to the extracellular matrix, cell signaling, oxidative stress, and innate and adaptive immunity have also been associated with POAG [7].

Two important research goals for POAG are the identification of persons at high risk for the disease and the advancement of neuroprotection for the optic nerve. Towards these goals, one key research aim is to identify systemic risk factors that can help predict POAG before vision loss occurs and potentially provide insight into the disease pathogenesis. One such marker may be microvascular hemorrhages identified using nailfold capillaroscopy [8, 9]. Nailfold capillaroscopy is a safe, noninvasive, and inexpensive clinical procedure that serves as a diagnostic tool in rheumatology and has been used to detect systemic microvascular abnormalities in POAG [9, 10]. Previous nailfold capillaroscopy studies have documented that hemorrhages, dilated capillaries, and avascular zones are increased in POAG compared with healthy control subjects [8, 9]. It remains unclear, however, whether this trend extends to high-risk ocular hypertension (OHT) patients or those with secondary forms of glaucoma.

The purpose of this study was to verify the specificity of nailfold capillary hemorrhages and other microvascular abnormalities as risk factors or predictors of POAG. Nailfold abnormalities were measured and compared between normal control subjects, POAG patients, OHT patients, and secondary angle-closure or angle-recession glaucoma (SG) patients. To examine the possibility that systemic microvascular abnormalities may be associated with IOP or the extent of visual field loss, subanalyses were conducted in POAG patients examining the association between nailfold capillary outcomes and previous glaucoma surgery, successful IOP control, or disease severity.

## 2. Materials and Methods

### 2.1. Study Population

All subjects were recruited from the Department of Ophthalmology, Northwestern University Feinberg School of Medicine, Chicago IL; the Division of Ophthalmology, John H. Stroger, Jr. Hospital of Cook County, Chicago IL; or the private practice of Zaparackas and Knepper Ltd. in Chicago, IL. In total, 569 participants were recruited from January 2014 to September 2019 including 277 control, 206 POAG, 29 SG, and 57 OHT subjects. This clinic-based, cross-sectional prospective study included a comprehensive ocular examination and nailfold capillaroscopy. All procedures were approved by the Institutional Review Boards of each study site. The study conformed to the tenets of Declaration of Helsinki, and all participants provided written informed consent.

All subjects were at least 35 years of age at the time of recruitment. Eyes with glaucoma had clinical findings consistent with glaucomatous optic neuropathy, including cup-disc ratio ≥0.6 and abnormal visual fields. Reliable visual field tests were acquired closest to the time of nailfold capillaroscopy using a Humphrey Field Analyzer (Carl Zeiss Meditec, Dublin, California, USA). Glaucoma severity was graded using visual field mean deviation scores from the more severely affected eye according to the Hodapp–Parrish–Anderson scale [11]. Early, intermediate, and late POAGs were defined as MD scores better than −6 dB, between −6 and −12 dB, and worse than −12 dB, respectively. Eyes with NTG had signs of glaucomatous optic neuropathy and no history of IOP >21 mm Hg. Eyes with high-tension POAG (HTG) had signs of glaucomatous optic neuropathy with a highest known IOP >21 mm Hg. OHT was defined as untreated IOP >21 mm Hg without visual field loss or any signs of glaucomatous optic neuropathy. SG patients had a diagnosis of angle-closure (*n* = 20) or angle-recession (*n* = 9) glaucoma.

Exclusion criteria consisted of connective tissue disease including systemic sclerosis, systemic lupus erythematosus, Sjogren's syndrome, antiphospholipid syndrome, dermatomyositis, psoriasis, or rheumatoid arthritis as these conditions have been associated with abnormal nailfold capillary patterns [12]. No subjects had comorbid mental cognitive impairment, dementia, or age-related macular degeneration, which have been associated with nailfold capillary abnormalities [13–15]. Additional exclusion criteria included bleeding diathesis, current treatment with chemotherapy, and history of trauma to the fingers or hands.

### 2.2. Collection of Covariate Data

Covariate data from the date of capillaroscopy were extracted from the subjects' medical record for use in multivariable logistic regression. Demographic information included age, sex, and race/ethnicity. Ocular information included IOP, cup-disc ratio, visual acuity, refractive error, lens status, and previous glaucoma surgery. Surgical success in patients with previous glaucoma surgery was defined as IOP ≤15 mm Hg at the time of examination. Information regarding preexisting medical conditions included the presence of hypertension, diabetes, arthritis, hyperlipidemia, cardiovascular disease, and cancer as well as any family history of glaucoma. Information about the use of medications included use of antiplatelet or anticoagulant medications (aspirin, warfarin, clopidogrel, etc.) which may cause nailfold capillary hemorrhages.

### 2.3. Nailfold Capillaroscopy

Nailfold capillaroscopy was performed using a JH-1004 microscope (Jiangsu Jiahua Electronic Instrument Co., Jiangsu, China) set at ×280 magnification as previously described [9]. In brief, subjects were asked to remain in a seated position for 15 minutes at room temperature prior to recording in order to stabilize blood flow. Cedar oil was applied to the nailfold to enhance epidermal translucency and visualization of the nailfold microvasculature. Videos were recorded of the fourth and fifth digits on each subject's nondominant hand to minimize confounding microvascular disruptions and ensure a sufficient sample of capillaries. Each video was 2 to 4 minutes in length and spanned the width of the capillary bed. Videos were analyzed by two masked graders who counted the total number of hemorrhages, dilated capillaries, and avascular zones for each participant. All values were normalized to counts per 100 capillaries based on the number of capillaries sampled to account for variability in capillary density. Hemorrhages were defined as extravascular deposits consisting of fresh, bright-red blood or old blood containing hemosiderin deposits. Dilated capillaries were defined as capillaries with a maximum width >50 *µ*m. Avascular zones were defined as horizontal regions ≥200 *µ*m displaying no capillaries.

### 2.4. Statistical Analysis

Statistical analysis was performed using Minitab 19 statistical software (State College, PA, US). Continuous demographic and clinical features were compared using unpaired *t*-tests. Frequencies of univariate categorical variables were compared between groups using *χ*^2^ tests. The mean numbers of hemorrhages, dilated capillaries, and avascular zones per 100 capillaries were compared using nonparametric Mann–Whitney *U* tests. Mantel–Haenszel *χ*^2^ tests were used to determine whether ordinal categories of nailfold findings were associated with case status.

To further assess associations between nailfold capillary abnormalities and case status, univariate and multivariable-adjusted logistic regression analyses were conducted with odds ratios (ORs) and 95% confidence intervals (CIs) calculated for each analysis. In the multivariable-adjusted model, adjustments were made for age, sex, race, study site, family history of glaucoma, hypertension, diabetes, non-skin cancer malignancy, and use of antiplatelet or anticoagulant medication. For each nailfold capillaroscopy finding, associations between binary values (e.g., any versus no hemorrhages) as well as between ordinal categories of values (“1” for 0 hemorrhages per 100 capillaries, “2” for >0 and <1, “3” for ≥1 and <2, and “4” for ≥(2) were evaluated between groups. All statistical tests were two sided with significance levels set at *P* < 0.05.

Interrater and intrarater reliability were calculated using weighted Cohen's kappa statistics accounting for ordinal categorical variables (*n* = 50). Agreement was high between graders for both hemorrhages (OR = 0.91, *P* < 0.0001) and all capillary abnormalities (OR = 0.89, *P* < 0.0001). Repeatability within graders was also high for both hemorrhages (OR = 0.96, *P* < 0.0001) and all capillary abnormalities (OR = 0.90, *P* < 0.0001).

## 3. Results

A total of 277 control, 206 POAG, 57 OHT, and 29 SG patients were included in this study. POAG patients were significantly older, more likely to be female, and more likely to be African-American compared with control subjects (Table 1). POAG patients also had a higher frequency of arthritis, diabetes, cardiovascular disease, and family history of glaucoma. The 206 POAG patients enrolled consisted of 173 (84.0%) with highest known IOP ≤21 mm Hg (HTG) and 33 (16.0%) with highest known IOP <21 mm Hg (NTG). HTG subjects were more likely than controls to have hypertension, diabetes, arthritis, cardiovascular disease, and a family history of glaucoma, whereas none of these variables were associated with NTG (Supplementary Table 1). No other demographic or clinical features differed between any subject type and controls.

The number of capillaries counted was variable within groups but lower in POAG and SG than controls and OHT (Supplementary Table 2). The higher age of POAG and SG patients could account for this difference; however, the correlation between age and capillaries counted was very weak (0.02). Because it is possible that factors such as video quality and natural variability in capillary density may influence the number of capillaries counted, the numbers of hemorrhages, dilated capillaries, and avascular zones were normalized to counts per 100 capillaries for all analyses. The effect of this normalization on hemorrhages is shown in Supplementary Table 2.

In the descriptive analysis of means (Table 2), POAG patients had significantly more hemorrhages (*P* < 0.0001), dilated capillaries (*P*=0.002), and avascular zones (*P*=0.0005) compared with controls. POAG patients also had significantly more hemorrhages than both SG (*P*=0.0007) and OHT (*P* < 0.0001) patients. There were no differences between HTG and NTG patients, POAG patients who had undergone glaucoma surgery and those who had not, or POAG patients with successful versus unsuccessful glaucoma surgery. OHT patients had more dilated capillaries than controls (*P*=0.04), whereas there was no difference in any measure between SG and controls.

In the descriptive frequency analysis (Table 3), the presence of one or more nailfold microvascular abnormalities was significantly higher in POAG than in controls: 85.9% versus 43.7% had at least one hemorrhage (*P* < 0.0001); 47.6% versus 33.2% had at least one dilated capillary (*P* < 0.0001); and 19.4% versus 8.7% had at least one avascular zone (*P*=0.001). In diagnostic terms, the presence of any hemorrhage in POAG yielded a sensitivity of 0.86, specificity of 0.54, and accuracy of 0.70. The presence of any of the three nailfold capillary abnormalities yielded a sensitivity of 0.95, specificity of 0.40, and accuracy of 0.67. In OHT, the presence of one or more hemorrhages was moderately more frequent compared with controls (59.6% versus 43.7%, *P*=0.03), although POAG patients were significantly more likely to have one or more hemorrhages compared with both OHT (*P* < 0.0001) and SG (*P*=0.001). When we evaluated the relationship between ordinal categories of abnormalities and subject type, there were significant associations between hemorrhages (*P* < 0.0001), dilated capillaries (*P*=0.03), and avascular zones (*P*=0.0007) in POAG compared with controls (Table 3). These trends were preserved when POAG was classified as HTG and NTG with the exception of dilated capillaries, which were associated with NTG (*P*=0.01) but not HTG (*P*=0.10).

In the univariate analysis in relation to control subjects, the presence of any hemorrhage (OR = 7.9, *P* < 0.0001) and increasing numbers of hemorrhages (OR = 14.2, *P* < 0.0001) were strongly associated with POAG (Table 4). There was a significant association between increasing hemorrhages and OHT compared with controls (OR = 1.9; *P*=0.006) and no association between any nailfold microvascular abnormalities and SG. In POAG, the association with increasing hemorrhages was significantly greater compared with both SG (*P*=0.006) and OHT (*P* < 0.0001), with the odds of two or more hemorrhages being 7.5 and 15.7 times greater in POAG compared with SG and OHT, respectively (Table 5).

Compared with the univariate analysis, the multivariable-adjusted model showed stronger associations between all microvascular abnormalities and POAG (Table 6). The presence of any hemorrhage (*P* < 0.0001), dilated capillary (*P* < 0.0001), or avascular zone (*P* < 0.0001) was significantly more likely to occur in POAG compared with controls. Likewise, there were significant associations between severity of hemorrhages (*P* < 0.0001), dilated capillaries (*P*=0.002), and avascular zones (*P*=0.002) and odds of POAG compared with controls, with POAG patients having 18.2 times greater odds of exhibiting two or more hemorrhages. OHT was associated with the presence of one or more hemorrhages (OR = 1.4, *P*=0.006) and increasing numbers of hemorrhages (*P*=0.01) compared with controls. SG was not associated with any nailfold abnormalities. When POAG was compared with OHT using the multivariable-adjusted model (Table 5), the odds of one or more hemorrhages (OR = 3.5, *P*=0.001) and the association between increasing numbers of hemorrhages (OR = 11.8, *P* < 0.0001) was greater in POAG. Likewise, when POAG was compared with SG, the odds of one or more hemorrhages (OR = 5.1, *P*=0.001) and the association between increasing numbers of hemorrhages was greater in POAG (OR = 9.1, *P*=0.001).

To examine the association between IOP and nailfold microvascular abnormalities in POAG, univariate and multivariable-adjusted logistic regression was performed in POAG patients examining the association between nailfold microvascular abnormalities and IOP, previous glaucoma surgery, or surgical success in those who had undergone surgery (Table 7). There were no significant differences between POAG patients who had undergone IOP-reducing surgery and those who had not. Among those who had undergone surgery, there were no differences between patients whose surgery was classified as successful versus unsuccessful. Additionally, there were no significant correlations between IOP and hemorrhages, dilated capillaries, or avascular zones in any group (Supplementary Table 3).

To evaluate the influence of POAG severity, nailfold capillary abnormalities were compared between POAG patients with early and intermediate-to-late stage disease (Supplementary Table 4). The presence of one or more hemorrhages was significantly more common in intermediate-to-late POAG in the univariate analysis (OR = 2.5, *P*=0.03) but not after adjustments in the multivariable model (OR = 2.2, *P*=0.08), suggesting that other factors such as age or comorbidities may influence the difference between groups. There was, however, a significant correlation between visual field loss and hemorrhages across all POAG patients (*P*=0.03) (Supplementary Table 3).

## 4. Discussion

In this study, POAG patients exhibited increased levels of nailfold hemorrhages, dilated capillaries, and avascular zones. The presence of any hemorrhage or 2 or more hemorrhages was found to be highly significant risk factors for POAG. OHT patients exhibited a trend towards increased hemorrhages characterized by moderate frequency but low severity, and SG patients exhibited no significant microvascular abnormalities. Compared directly with OHT and SG, hemorrhages were both more frequent and more severe in POAG. No associations were found between nailfold capillary abnormalities and IOP, glaucoma surgery, or surgical success in POAG, and hemorrhages were only moderately associated with disease severity.

Studies demonstrating systemic findings in POAG have reported an increased prevalence of diabetes [2], cardiovascular system irregularities such as vasospasm [10, 16], altered hemorheological properties such as increased platelet activation/aggregation [17–21], and reduced cerebrospinal fluid pressure [22]. Additionally, POAG has been associated with alterations in systemic blood flow (both high and low blood pressure) and ocular perfusion pressure [23]. These properties suggest a possible vascular etiology to the development and progression of POAG. Vascular alterations such as vasospasm are associated with altered ocular blood flow and consequently decreased autoregulation of ocular perfusion pressure [10]. Decreased autoregulation of ocular blood flow and ocular perfusion pressure have been demonstrated in POAG [10, 24, 25] and can lead to ischemia, hypoxia, and ultimately retinal ganglion cell death. There have also been associations reported between optic disc hemorrhages and both nailfold hemorrhages and avascular zones in POAG [8], suggesting a comparable microvascular disruption at the systemic and ocular levels. Nailfold capillaroscopy may provide an easily accessible method of assessing risk of POAG based on systemic microvascular manifestations.

The exact cause of nailfold microvascular abnormalities in POAG remains unknown. An increased number of extravascular hemorrhages suggest a defect in the capillary wall and potentially present an opportunity for intervention. The endothelial cells lining the capillary wall are joined at their borders, a thin basal lamina, and are not supported by an outer tunic of smooth muscle cells. The luminal diameter ranges from 3 to 10 *μ*m, and blood flow is regulated by pericapillary contractile cells called pericytes. The rate of capillary endothelial replication is age-dependent and may have a half-life of one to three years [26]. In addition, circulating endothelial progenitor cells normally recruited to the capillary wall to maintain the endothelial barrier function are reduced in POAG [27]. Endothelial progenitor cell enrichment therapy has thus been suggested as a measure to target ischemic endothelial damage in POAG [28]. Other environmental cues such as the extracellular matrix and basal lamina also influence capillary function and may play a role in nailfold capillary abnormalities.

The elevated risk of POAG associated with OHT [29] makes the evaluation of nailfold capillaroscopy in these subjects, which has never previously been reported, particularly intriguing. The fact that hemorrhage prevalence increased in a stepwise fashion from control (44%) to OHT (60%) to POAG (86%) could be explained by this increased risk. Combined with the finding that 81% of early-stage POAG patients already exhibited hemorrhages, these results strongly suggest that nailfold hemorrhages occur early in the majority of POAG of cases and likely precede measurable changes in functional and structural diagnostic criteria such as visual field, cup-disc ratio, and retinal nerve fiber layer thickness. Interestingly, OHT status was more predictive of hemorrhages than IOP itself, suggesting that hemorrhages may be useful in predicting which OHT patients progress to POAG and which do not. This study was cross sectional in design; however, longitudinal studies would be needed to fully evaluate the time course of nailfold microvascular abnormalities in relation to POAG and the association between those abnormalities and risk of progression in controls and OHT patients.

Despite being sufficient in power analyses, we recognize that some groups consisted of disproportionate sample sizes and that this may have limited the reliability of certain comparisons. Specifically, there was a comparably low number of SG patients enrolled and a high ratio of HTG to NTG patients in the POAG sample. Future studies would need to specifically target the less-common SG population.

Nailfold hemorrhages measured using nailfold capillaroscopy appear to be a fast, easy, and reliable clinical risk factor for POAG. A large majority of early-stage POAG patients and nearly all intermediate/late-stage POAG patients exhibit hemorrhages, which do not appear to be associated with known risk factors such as IOP or common comorbidities such as hypertension. In contrast, the evaluation of other reported systemic vascular risk factors for the disease may be costly and require elaborate and time-consuming procedures, limiting their usefulness in a routine clinical setting. Nailfold capillaroscopy may serve not only as a useful assessment of risk in POAG but also as a research tool facilitating the identification of disease mechanisms and potential therapeutic targets.

## 5. Conclusions

In conclusion, POAG patients exhibited increased levels of all nailfold microvascular abnormalities measured. Nailfold hemorrhages, in particular, may serve as a useful clinical risk factor for the development and/or progression of POAG.

## Figures and Tables

**Table 1:** Demographic and clinical features of the study cohort.

	Control (*n* = 277)	POAG (*n* = 206)	*P* value vs. control	SG (*n* = 29)	*P* value vs. control	OHT (*n* = 57)	*P* value vs. control
Age in years, mean (SD)	63.2 (10.8)	67.5 (10.6)	<0.0001^*∗*^	65.0 (11.2)	0.40^*∗*^	62.5 (12.5)	0.68^*∗*^
Sex, *n* (%)							
Female	157 (56.7)	95 (46.1)	0.02	18 (62.1)	0.42	36 (63.2)	0.36
Male	120 (43.3)	111 (53.9)		11 (37.9)		21 (36.8)	
Race/ethnicity, *n* (%)							
Caucasian	169 (61.0)	88 (42.7)	<0.0001	9 (33.3)	0.002	39 (68.4)	0.29
African-American	59 (21.3)	95 (46.1)		15 (55.6)		12 (21.1)	
Asian/Pacific Islander	7 (2.5)	11 (5.3)		1 (3.7)		3 (5.3)	
Hispanic	42 (15.2)	12 (5.8)		4 (14.8)		3 (5.3)	
Any cancer, *n* (%)	30 (10.8)	20 (9.7)	0.69	3 (10.3)	0.94	3 (5.3)	0.17
Non-skin cancer malignancy, *n* (%)	19 (6.9)	19 (9.2)	0.35	2 (6.9)	0.99	1 (1.8)	0.09
Anticoagulant medication, *n* (%)	69 (24.9)	46 (22.3)	0.51	5 (17.2)	0.36	9 (15.8)	0.13
Cataract, *n* (%)	90 (32.5)	70 (34.0)	0.73	6 (20.7)	0.19	16 (28.1)	0.51
Cataract surgery, *n* (%)	27 (1.0)	26 (12.6)	0.33	1 (3.4)	0.26	6 (10.5)	0.87
Arthritis, *n* (%)	50 (18.1)	19 (9.2)	0.005	1 (3.4)	0.05	6 (10.5)	0.15
Hypertension, *n* (%)	116 (41.9)	105 (51.0)	0.05	9 (31.0)	0.26	23 (40.4)	0.83
Diabetes, *n* (%)	58 (20.9)	65 (31.6)	0.008	5 (17.2)	0.64	12 (22.8)	0.76
Hyperlipidemia, *n* (%)	61 (22.0)	42 (20.4)	0.66	7 (24.1)	0.79	15 (26.3)	0.49
Cardiovascular disease, any, *n* (%)	30 (10.8)	34 (16.5)	0.05	1 (3.4)	0.21	5 (8.8)	0.70
Family history of glaucoma, *n* (%)	57 (20.6)	76 (36.9)	<0.0001	7 (24.1)	0.65	13 (22.8)	0.71
IOP (mm Hg), mean (SD)	14.6 (3.5)	17.2 (6.7)	<0.0001^*∗*^	20.4 (8.2)	<0.0001^*∗*^	19.4 (4.2)	<0.0001^*∗*^
Disease severity, *n* (%)							
Early	———	111 (53.9)		———		———	
Intermediate	———	48 (23.3)		———		———	
Late	———	47 (22.8)		———		———	

^*∗*^the *t*-test; all other *P* values, the *χ*2 test. HTG, high-tension glaucoma; IOP, intraocular pressure; NTG, normal-tension glaucoma; OHT, ocular hypertension; POAG, primary open-angle glaucoma; SG, secondary glaucoma.

**Table 2:** Mean number of nailfold capillary abnormalities per 100 capillaries.

Subject type	*n*	Hemorrhages/100				Dilated capillaries/100				Avascular zones/100			
		Mean	*SD*	*P* value^*∗*^	*P* value^†^	Mean	*SD*	*P* value^*∗*^	*P* value^†^	Mean	*SD*	*P* value^*∗*^	*P* value^†^
**Control**	277	0.77	1.39			0.69	1.37			0.09	0.34		
**POAG**	206	1.88	1.68	<0.0001		1.02	1.55	0.002		0.20	0.47	0.0005	
High-tension	173	1.87	1.71	<0.0001		1.00	1.63	0.02		0.19	0.47	0.003	
Normal-tension	33	1.91	1.60	<0.0001		1.10	1.05	0.0007		0.24	0.44	0.001	
No surgery	151	1.87	1.82	<0.0001		0.98	1.62	0.03		0.53	0.53	0.89	
Surgery	55	1.88	1.44	<0.0001		1.05	1.68	0.005		0.29	0.29	<0.0001	
Successful	19	2.05	1.57	<0.0001		1.24	1.69	0.06		0.13	0.32	0.38	
Unsuccessful	36	2.00	1.70	<0.0001		0.94	1.69	0.21		0.07	0.28	0.60	
**SG**	29	0.89	0.93	0.08	0.0007	0.47	0.82	0.87	0.12	0.09	0.34	0.78	0.12
**OHT**	57	0.87	1.19	0.09	<0.0001	0.98	1.36	0.04	0.95	0.16	0.44	0.11	0.48

^*∗*^the Mann–Whitney *U* test versus control. ^†^the Mann–Whitney *U* test versus POAG. HTG, high-tension glaucoma; NTG, normal-tension glaucoma; OHT, ocular hypertension; POAG, primary open-angle glaucoma; SG, secondary glaucoma.

**Table 3:** Descriptive analyses of nailfold capillary abnormalities.

Nailfold microvascular feature	Control	POAG (*n* = 206)		HTG (*n* = 173)		NTG (*n* = 33)		SG (*n* = 29)		*P* value vs. POAG	OHT (*n* = 57)		*P* value vs. POAG
	*n* (%)	*n* (%)	*P* value vs. control	*n* (%)	*P* value vs. control	*n* (%)	*P* value vs. control	*n* (%)	*P* value vs. control		*n* (%)	*P* value vs. control	
**Hemorrhages**													
**/100 capillaries, *n* (%)**													
0.0	156 (56.3)	29 (14.1)	<0.0001^*∗*^	24 (13.9)	<0.0001^*∗*^	5 (15.2)	<0.0001^*∗*^	11 (37.9)	0.49^*∗*^	0.001^*∗*^	23 (40.4)	0.96^*∗*^	<0.0001^*∗*^
>0.0 and <1.0	58 (20.9)	57 (27.7)		50 (28.9)		7 (21.2)		9 (31.0)			18 (31.6)		
≥1.0 and <2.0	32 (11.6)	41 (19.9)		34 (19.7)		7 (21.2)		10 (34.5)			12 (21.1)		
≥2.0	31 (11.2)	79 (38.3)		65 (37.6)		12 (42.4)		4 (13.8)			4 (7.0)		
Any hemorrhages	121 (43.7)	177 (85.9)	<0.0001^†^	149 (86.1)	<0.0001^†^	28 (84.8)	<0.0001^†^	18 (62.1)	0.06^†^	0.001^†^	34 (59.6)	0.03^†^	<0.0001^†^

**Dilated capillaries**													
**/100 capillaries, *n* (%)**													
0.0	185 (66.8)	108 (52.4)	0.03^*∗*^	97 (56.1)	0.10	11 (33.3)	0.01^*∗*^	19 (65.5)	0.59^*∗*^	0.11^*∗*^	30 (52.6)	0.14^*∗*^	0.92^*∗*^
>0.0 and <1.0	32 (11.6)	36 (17.5)		28 (16.2)		8 (24.2)		7 (24.1)			8 (14.0)		
≥1.0 and <2.0	25 (9.0)	24 (11.7)		18 (10.4)		6 (18.2)		5 (17.2)			8 (14.0)		
≥2.0	32 (12.6)	38 (18.4)		30 (17.3)		8 (24.2)		2 (6.9)			11 (19.3)		
Any dilated capillaries	92 (33.2)	98 (47.6)	<0.0001^†^	76 (43.9)	0.02^†^	22 (66.7)	<0.0001^†^	10 (34.5)	0.89^†^	0.19^†^	27 (47.4)	0.05^†^	0.05^†^

**Avascular zones**													
**/100 capillaries, *n* (%)**													
0.0	253 (91.3)	166 (80.6)	0.0007^*∗*^	142 (82.1)	0.002^*∗*^	24 (72.7)	0.03^*∗*^	27 (93.1)	0.49^*∗*^	0.45^*∗*^	48 (84.2)	0.21^*∗*^	0.29^*∗*^
>0.0 and <0.75	11 (4.0)	12 (5.8)		8 (4.6)		4 (12.1)		0 (0.0)			5 (8.8)		
≥0.75	13 (4.7)	28 (13.6)		23 (13.3)		5 (15.2)		2 (6.9)			4 (7.0)		
Any avascular zones	24 (8.7)	40 (19.4)	0.001^†^	31 (17.9)	0.004^†^	9 (27.3)	0.004^†^	2 (6.9)	0.98^†^	0.10^†^	9 (15.8)	0.12^†^	0.53^†^

^*∗*^the Mantel–Haenszel–Cochran test. ^†^ the *χ*^2^ test. HTG, high-tension glaucoma; NTG, normal-tension glaucoma; OHT, ocular hypertension; POAG, primary open-angle glaucoma; SG, secondary glaucoma.

**Table 4:** Univariate logistic regression analysis of nailfold capillary abnormalities in relation to control subjects.

Nailfold microvascular feature	POAG (*n* = 206)		HTG (*n* = 173)		NTG (*n* = 33)		SG (*n* = 29)		OHT (*n* = 57)	
	OR (95% CI)	*P* value	OR (95% CI)	*P* value	OR (95% CI)	*P* value	OR (95% CI)	*P* value	OR (95% CI)	*P* value
Hemorrhages/100 capillaries										
0.0	1.0 (ref)		1.0 (ref)		1.0 (ref)		1.0 (ref)		1.0 (ref)	
>0.0 and <1.0	5.3 (3.1–9.1)	<0.0001	5.6 (3.2–9.9)	<0.0001	3.8 (1.1–12.3)	<0.0001	2.2 (0.9–5.6)	0.30	2.1 (1.1–4.2)	0.05
≥1.0 and <2.0	6.7 (3.6–12.3)		6.7 (3.5–12.7)		6.6 (2.0–22.1)		2.1 (0.7–6.6)		2.5 (1.1–5.4)	
≥2.0	14.2 (7.9–25.2)		14.1 (7.7–25.9)		14.6 (4.9–43.4)		1.9 (0.6–6.3)		0.9 (0.3–2.8)	
Any hemorrhages	7.9 (5.0–12.5)	<0.0001	8.0 (4.9–13.1)	<0.0001	7.2 (2.7–19.3)	<0.0001	2.1 (1.0–4.6)	0.06	1.9 (1.1–3.4)	0.03

Dilated capillaries/100 capillaries										
0.0	1.0 (ref)		1.0 (ref)		1.0 (ref)		1.0 (ref)		1.0 (ref)	
>0.0 and <1.0	1.9 (1.1–3.3)	0.02	1.7 (0.9–2.9)	0.14	4.2 (1.6–11.3)	0.002	2.1 (0.8–5.5)	0.20	1.5 (0.6–3.7)	0.23
≥1.0 and <2.0	1.8 (1.0–3.2)		1.4 (0.7–2.6)		5.4 (2.0–14.7)		0.4 (0.1–3.0)		2.0 (0.8–4.8)	
≥2.0	1.8 (1.0–3.0)		1.6 (0.9–2.8)		2.9 (1.0–8.3)		0.6 (0.1–2.5)		1.9 (0.9–4.2)	
Any dilated capillaries	1.8 (1.3–2.6)	0.001	1.6 (1.1–2.3)	0.02	4.0 (1.9–8.6)	<0.0001	1.1 (0.5–2.4)	0.89	1.8 (1.0–3.2)	0.05

Avascular zones/100 capillaries										
0.0	1.0 (ref)		1.0 (ref)		1.0 (ref)		1.0 (ref)		1.0 (ref)	
>0.0 and <0.75	1.7 (0.7–3.9)	0.001	1.3 (0.5–3.3)	0.005	3.8 (1.1–13.0)	0.02	---	---	2.4 (0.8–7.2)	0.27
≥0.75	3.3 (1.7–6.5)		3.2 (1.5–6.4)		4.1 (1.3–12.3)		---		1.6 (0.5–5.2)	
Any avascular zones	2.5 (1.5–4.4)	0.001	2.3 (1.3–4.1)	0.004	4.0 (1.7–9.5)	0.004	0.8 (0.2–3.5)	0.74	2.0 (0.9–4.5)	0.12

CI, confidence interval; HTG, high-tension glaucoma; NTG, normal-tension glaucoma; OHT, ocular hypertension; OR, odds ratio; POAG, primary open-angle glaucoma; SG, secondary glaucoma.

**Table 5:** Univariate and multivariable-adjusted logistic regression analysis of nailfold capillary abnormalities in relation to POAG compared with SG and OHT.

Nailfold microvascular feature	POAG (*n* = 206) vs. SG (*n* = 29)			POAG (*n* = 206) vs. OHT (*n* = 57)		
	Univariate OR (95% CI)	Multivariate OR (95% CI)	*P* value	Univariate OR (95% CI)	Multivariate OR (95% CI)	*P* value
Hemorrhages/100 capillaries						
0.0	1.0 (ref)			1.0 (ref)		
>0.0 and <1.0	2.4 (0.9–6.5)	3.2 (1.1–9.7)	0.004	2.5 (1.2–5.4)	2.0 (0.8–4.9)	<0.0001
≥1.0 and <2.0	3.1 (1.0–9.9)	5.1 (1.4–18.5)		2.7 (1.2–6.3)	2.7 (1.1–6.8)	
≥2.0	7.5 (2.2–25.4)	9.1 (2.5–33.7)		15.7 (5.0–49.2)	11.8 (3.5–39.5)	
Any hemorrhages	3.7 (1.6–8.7)	5.1 (1.9–13.4)	0.001	4.1 (2.1–7.8)	3.5 (1.7–7.3)	0.001

Dilated capillaries/100 capillaries						
0.0	1.0 (ref)			1.0 (ref)		
>0.0 and <1.0	0.9 (0.4–2.3)	1.1 (0.4–3.0)	0.18	1.3 (0.5–3.0)	1.9 (0.7–5.0)	0.53
≥1.0 and <2.0	4.2 (0.5–33.1)	4.5 (0.6–36.8)		0.8 (0.3–2.0)	1.0 (0.3–2.8)	
≥2.0	3.3 (0.7–15.0)	3.2 (0.7–15.8)		1.0 (0.4–2.1)	1.5 (0.6–3.9)	
Any dilated capillaries	1.7 (0.8–3.9)	1.9 (0.8–4.5)	0.15	1.0 (0.6–1.8)	1.1 (1.0–1.1)	0.28

Avascular zones/100 capillaries						
0.0	1.0 (ref)			1.0 (ref)		
>0.0 and <0.75	———	———	0.08	0.7 (0.2–2.1)	0.8 (0.2–2.6)	0.29
≥0.75	3.3 (0.7–14.3)	3.3 (0.7–15.4)		2.0 (0.7–6.1)	2.3 (0.7–7.6)	
Any avascular zones	3.3 (0.7–14.3)	3.3 (0.7–15.4)	0.08	0.3 (0.6–2.8)	1.4 (0.6–3.5)	0.41

The model adjusts for age (in years), sex, race, family history of glaucoma, hypertension, use of antiplatelet medication, and study site. CI, confidence interval; OHT, ocular hypertension; OR, odds ratio; POAG, primary open-angle glaucoma; SG, secondary glaucoma.

**Table 6:** Multivariable-adjusted logistic regression analysis of nailfold capillary abnormalities in relation to control subjects.

Nailfold microvascular feature	All POAG (*n* = 206)		HTG (*n* = 173)		NTG (*n* = 33)		SG (*n* = 29)		OHT (*n* = 57)	
	OR (95% CI)	*P* value	OR (95% CI)	*P* value	OR (95% CI)	*P* value	OR (95% CI)	*P* value	OR (95% CI)	*P* value
Hemorrhages/100 capillaries										
0.0	1.0 (ref)		1.0 (ref)		1.0 (ref)		1.0 (ref)		1.0 (ref)	
>0.0 and <1.0	5.6 (2.8–11.0)	<0.0001	6.5 (3.0–14.0)	<0.0001	4.6 (1.2–18.1)	<0.0001	1.4 (0.4–4.6)	0.65	2.7 (1.3–5.8)	0.01
≥1.0 and <2.0	6.7 (3.1–14.6)		7.8 (3.3–18.3)		5.8 (1.3–25.5)		2.4 (0.6–8.9)		3.3 (1.4–8.2)	
≥2.0	18.3 (8.5–39.4)		18.7 (3.3–18.3)		19.0 (5.0–72.0)		1.2 (0.3–5.3)		1.0 (0.3–3.5)	
Any hemorrhages	8.3 (4.6–15.0)	<0.0001	9.1 (4.7–17.8)	<0.0001	8.2 (2.6–26.3)	<0.0001	1.6 (0.6–4.1)	0.34	1.4 (0.7–2.8)	0.006

Dilated capillaries/100 capillaries										
0.0	1.0 (ref)		1.0 (ref)		1.0 (ref)		1.0 (ref)		1.0 (ref)	
>0.0 and <1.0	2.5 (1.3–4.9)	0.002	2.3 (1.1–5.0)	0.02	3.4 (1.0–11.2)	0.01	2.5 (0.8–8.0)	0.46	1.3 (0.5–3.4)	0.26
≥1.0 and <2.0	2.0 (0.9–4.5)		1.4 (0.6–3.5)		5.1 (1.4–17.9)		0.6 (0.1–6.8)		2.0 (0.7–5.2)	
≥2.0	3.1 (1.5–6.1)		2.9 (1.3–6.4)		4.7 (1.4–15.7)		1.1 (0.2–6.1)		2.2 (0.9–5.2)	
Any dilated capillaries	2.5 (1.6–4.2)	<0.0001	2.2 (1.3–3.9)	0.003	4.2 (1.7–10.6)	0.001	1.6 (0.6–4.2)	0.36	1.8 (0.9–3.4)	0.08

Avascular zones/100 capillaries										
0.0	1.0 (ref)		1.0 (ref)		1.0 (ref)		1.0 (ref)		1.0 (ref)	
>0.0 and <0.75	3.2 (1.1–9.2)	0.002	2.6 (0.7–9.4)	0.006	5.6 (1.3–24.0)	0.05	———	0.46	4.5 (1.4–15.0)	0.05
≥0.7	4.0 (1.6–9.9)		4.6 (1.7–12.6)		3.3 (0.8–14.1)		2.3 (0.3–18.0)		2.0 (0.6–7.2)	
Any avascular zones	3.7 (1.8–7.6)	<0.0001	3.7 (1.6–8.5)	0.002	4.2 (1.3–13.1)	0.02	2.3 (0.3–18.0)	0.46	3.0 (1.2–7.6)	0.03

The model adjusts for age (in years), sex, race, family history of glaucoma, hypertension, use of antiplatelet medication, and study site. CI, confidence interval; HTG, high-tension glaucoma; NTG, normal-tension glaucoma; OHT, ocular hypertension; OR, odds ratio; POAG, primary open-angle glaucoma; SG, secondary glaucoma.

**Table 7:** Multivariable-adjusted logistic regression analysis of nailfold capillary abnormalities in relation to IOP (mm Hg), previous glaucoma surgery, and surgical outcomes in POAG patients.

Nailfold microvascular feature	IOP >15 (*n* = 129) vs. IOP ≤15 (*n* = 77)		Previous surgery (*n* = 55) vs. no surgery (*n* = 118)		Successful (*n* = 19) vs. unsuccessful (*n* = 36) surgery	
	OR (95% CI)	*P* value	OR (95% CI)	*P* value	OR (95% CI)	*P* value
Hemorrhages/100 capillaries						
0.0	1.0 (ref)		1.0 (ref)		1.0 (ref)	
>0.0 and <1.0	1.7 (0.6–4.8)	0.07	0.6 (0.2–2.0)	0.42	3.3 (0.1–80.1)	0.17
≥1.0 and <2.0	0.7 (0.2–1.9)		1.4 (0.4–4.8)		1.2 (0.0–33.9)	
≥2.0	0.6 (0.2–1.6)		0.9 (0.3–2.7)		7.4 (0.3–178.3)	
Any hemorrhages	0.8 (0.3–2.0)	0.71	0.9 (0.3–2.5)	0.80	3.4 (0.2–60.4)	0.37

Dilated capillaries/100 capillaries						
0.0	1.0 (ref)		1.0 (ref)		1.0 (ref)	
>0.0 and <1.0	1.1 (0.5–2.6)	0.99	2.2 (0.9–5.4)	0.38	1.0 (0.1–8.1)	0.06
≥1.0 and <2.0	1.0 (0.4–2.6)		1.4 (0.5–4.1)		5.0 (0.8–32.9)	
≥2.0	0.9 (0.4–2.2)		1.5 (0.5–4.4)		13.0 (0.9–192.1)	
Any dilated capillaries	1.0 (0.5–1.9)	0.96	1.7 (0.9–3.5)	0.12	3.6 (0.9–14.8)	0.08

Avascular zones/100 capillaries						
0.0	1.0 (ref)		1.0 (ref)		1.0 (ref)	
>0.0 and <0.75	1.1 (0.3–4.4)	0.67	0.3 (0.0–2.6)	0.23	———	0.27
≥0.75	0.7 (0.3–1.6)		0.5 (0.1–1.6)		3.3 (0.4–28.4)	
Any avascular zones	0.8 (0.4–1.7)	0.54	0.4 (0.1–1.2)	0.09	3.3 (0.4–28.4)	0.27

The model adjusts for age (in years), sex, race, family history of glaucoma, hypertension, use of antiplatelet medication, and study site. CI, confidence interval; IOP, intraocular pressure; OR, odds ratio; POAG, primary open-angle glaucoma; Ref, reference.

## Data Availability

The datasets used to support the findings of this study are available from the corresponding author upon request.

## Supplementary Materials

Supplementary Materials Supplementary Table 1: demographic and clinical features of POAG patients classified as high-tension (HTG) or normal-tension (NTG) glaucoma. Supplementary Table 2: mean numbers of capillaries counted and the effect of normalization to counts per 100 capillaries for hemorrhages. Supplementary Table 3: correlations between nailfold microvascular outcomes per 100 capillaries with IOP or visual field loss. Supplementary Table 4: univariate and multivariable-adjusted logistic regression analysis of nailfold capillary abnormalities in relation to intermediate-to-late (*n* = 95) vs. early POAG (*n* = 111).
