# Supplementary material for: Nailfold Capillary Hemorrhages: Microvascular Risk Factors for Primary Open-Angle Glaucoma
Source: J Ophthalmol. 2020 Jun 8;2020:8324319. doi: 10.1155/2020/8324319 (PMC7301193; doi:10.1155/2020/8324319)
Supplement: Supplementary Materials — Supplementary Table 1: demographic and clinical features of POAG patients classified as high-tension (HTG) or normal-tension (NTG) glaucoma. Supplementary Table 2: mean numbers of capillaries counted and the effect of normalization to counts per 100 capillaries for hemorrhages. Supplementary Table 3: correlations between nailfold microvascular outcomes per 100 capillaries with IOP or visual field loss. Supplementary Table 4: univariate and multivariable-adjusted logistic regression analysis of nailfold capillary abnormalities in relation to intermediate-to-late (n = 95) vs. early POAG (n = 111). [file 8324319.f1.docx]

Supplementary Table 1. Demographic and clinical features of POAG patients classified as high-tension (HTG) or normal-tension (NTG) glaucoma.

| **Demographic or clinical feature** | | **Control**  **(n=277)** | **HTG**  **(n=173)** | **P value vs Control** | **NTG**  **(n=33)** | **P value vs Control** |
| --- | --- | --- | --- | --- | --- | --- |
| Age in years, mean (SD) | | 63.2 (10.8) | 67.5 (10.8) | <0.0001* | 67.5 (9.7) | 0.03* |
| Sex, n (%) | |  |  |  |  |  |
|  | Female | 157 (56.7) | 76 (43.9) | 0.008 | 19 (57.6) | 0.92 |
|  | Male | 120 (43.3) | 97 (56.1) |  | 14 (42.4) |  |
| Race/ethnicity, n (%) | |  |  |  |  |  |
|  | Caucasian | 169 (61.0) | 67 (38.7) | <0.0001 | 21 (63.6) | 0.77 |
|  | African-American | 59 (21.3) | 89 (51.4) |  | 6 (18.2) |  |
|  | Asian/Pacific Islander | 7 (2.5) | 6 (3.5) |  | 5 (15.2) |  |
|  | Hispanic | 42 (15.2) | 11 (6.4) |  | 1 (3.0) |  |
| Any cancer, n (%) | | 30 (10.8) | 12 (8.1) | 0.34 | 6 (18.2) | 0.24 |
| Non-skin cancer malignancy, n (%) | | 19 (6.9) | 13 (7.5) | 0.81 | 6 (18.2) | 0.05 |
| Anticoagulant medication, n (%) | | 69 (24.9) | 36 (20.8) | 0.32 | 10 (30.3) | 0.51 |
| Cataract, n (%) | | 90 (32.5) | 58 (33.5) | 0.82 | 12 (36.4) | 0.66 |
| Cataract surgery, n (%) | | 27 (1.0) | 23 (13.3) | 0.25 | 3 (9.1) | 0.90 |
| Arthritis, n (%) | | 50 (18.1) | 17 (9.8) | 0.02 | 2 (6.1) | 0.05 |
| Hypertension, n (%) | | 116 (41.9) | 94 (54.3) | 0.01 | 11 (33.3) | 0.34 |
| Diabetes, n (%) | | 58 (20.9) | 59 (34.1) | 0.002 | 6 (18.2) | 0.71 |
| Hyperlipidemia, n (%) | | 61 (22.0) | 35 (20.2) | 0.65 | 7 (21.2) | 0.92 |
| Cardiovascular disease, n (%) | | 30 (10.8) | 30 (17.3) | 0.04 | 4 (12.1) | 0.78 |
| Family history of glaucoma, n (%) | | 57 (20.6) | 64 (37.0) | <0.0001 | 12 (36.4) | 0.05 |
| IOP (mm Hg), mean (SD) | | 14.6 (3.5) | 17.7 (7.4) | <0.0001* | 14.7 (3.3) | 0.85* |
| Disease severity, n (%) | |  |  |  |  |  |
|  | Mild | --- | 89 (51.4) |  | 22 (66.7) |  |
|  | Moderate | --- | 42 (24.3) |  | 6 (18.2) |  |
|  | Severe | --- | 42 (24.3) |  | 5 (15.2) |  |

*t-test; All other P values χ2 test. HTG, high-tension glaucoma; IOP, intraocular pressure; NTG, normal-tension glaucoma; POAG, primary open-angle glaucoma.

Supplementary Table 2. Mean numbers of capillaries counted and the effect of normalization to counts per 100 capillaries for hemorrhages.

| **Group** | **n** | **Capillaries,**  **Mean (SD)** | **P value*** | **Hemorrhages unadjusted,**  **Mean (SD)** | **P value^†^** | **Hemorrhages per 100, Mean (SD)** | **P value^†^** |
| --- | --- | --- | --- | --- | --- | --- | --- |
| Control | 277 | 130.1 (33.9) |  | 0.99 (1.71) |  | 0.77 (1.39) |  |
| POAG | 206 | 121.9 (23.6) | 0.003 | 2.30 (2.25) | <0.0001 | 1.88 (1.68) | <0.0001 |
| SG | 29 | 118.5 (16.2) | 0.07 | 1.10 (2.23) | 0.11 | 0.89 (0.93) | 0.08 |
| OHT | 57 | 130.6 (33.2) | 0.91 | 1.12 (1.59) | 0.09 | 0.87 (1.19) | 0.09 |

*t-test; ^†^Mann-Whitney U test versus Control. OHT, ocular hypertension; POAG, primary open-angle glaucoma; SG, secondary glaucoma.

Supplementary Table 3. Correlations between nailfold capillary abnormalities per 100 capillaries and IOP or visual field loss.

|  |  |  | **Hemorrhages** | | **Dilated capillaries** | | **Avascular zones** | |
| --- | --- | --- | --- | --- | --- | --- | --- | --- |
| **Variable** | **Group** | **n** | **Correlation coefficient*** | **P value** | **Correlation coefficient** | **P value** | **Correlation coefficient** | **P value** |
| IOP | Control | 277 | -0.079 | 0.19 | -0.030 | 0.58 | -0.032 | 0.59 |
|  | OHT | 57 | -0.048 | 0.73 | -0.089 | 0.51 | 0.014 | 0.92 |
|  | SG | 29 | 0.137 | 0.48 | -0.247 | 0.20 | 0.102 | 0.60 |
|  | POAG | 206 | -0.083 | 0.23 | -0.026 | 0.71 | -0.053 | 0.45 |
|  |  |  |  |  |  |  |  |  |
| Visual field loss | SG | 29 | 0.092 | 0.64 | -0.301 | 0.11 | 0.348 | 0.06 |
|  | POAG | 206 | 0.155 | 0.03 | -0.039 | 0.58 | -0.027 | 0.70 |

*Spearman's rank-order correlation coefficient. IOP, intraocular pressure; OHT, ocular hypertension; POAG, primary open-angle glaucoma; SG, secondary glaucoma

Supplementary Table 4. Univariate and multivariable-adjusted logistic regression analysis of nailfold capillary abnormalities in relation to moderate-to-severe (n = 95) vs. early POAG (n = 111).

| **Nailfold microvascular**  **feature** | | **Univariate model** | | **Multivariable-adjusted model*** | |
| --- | --- | --- | --- | --- | --- |
|  |  | **OR (95% CI)** | **P value** | **OR (95% CI)** | **P value** |
| **Hemorrhages / 100 capillaries** | |  |  |  |  |
|  | 0.0 | 1.0 (ref) |  | 1.0 (ref) |  |
|  | > 0.0 and < 1.0 | 2.1 (0.8-5.4) | 0.11 | 1.9 (0.7-5.3) | 0.32 |
|  | ≥ 1.0 and < 2.0 | 2.5 (0.9-7.0) |  | 2.2 (0.8-6.6) |  |
|  | ≥ 2.0 | 3.0 (1.2-7.5) |  | 2.4 (0.9-6.4) |  |
|  | Any hemorrhages | 2.5 (1.1-6.0) | 0.03 | 2.2 (0.9-5.5) | 0.08 |
| **Dilated capillaries / 100 capillaries** | |  |  |  |  |
|  | 0.0 | 1.0 (ref) |  | 1.0 (ref) |  |
|  | > 0.0 and < 1.0 | 0.8 (0.4-1.6) | 0.62 | 1.0 (0.5-2.3) | 0.92 |
|  | ≥ 1.0 and < 2.0 | 1.3 (0.5-3.1) |  | 1.3 (0.5-3.4) |  |
|  | ≥ 2.0 | 0.7 (0.3-1.5) |  | 0.9 (0.4-2.1) |  |
|  | Any dilated capillaries | 0.8 (0.5-1.5) | 0.54 | 1.1 (0.6-1.9) | 0.84 |
| **Avascular zones / 100 capillaries** | |  |  |  |  |
|  | 0.0 | 1.0 (ref) |  | 1.0 (ref) |  |
|  | > 0.0 and < 0.75 | 0.8 (0.2-2.6) | 0.88 | 1.0 (0.3-3.4) | 0.95 |
|  | ≥ 0.75 | 0.8 (0.4-1.9) |  | 1.1 (0.5-2.7) |  |
|  | Any avascular zones | 0.8 (0.4-1.7) | 0.61 | 1.1 (0.5-2.3) | 0.83 |

*Model adjusts for age, sex, race, family history of glaucoma, hypertension, use of antiplatelet medication, and study site. CI., Confidence interval; OR, odds ratio; POAG, primary open-angle glaucoma; ref, reference.
